# Supplementary material for: Sorting at embryonic boundaries requires high heterotypic interfacial tension
Source: Nat Commun. 2017 Jul 31;8:157. doi: 10.1038/s41467-017-00146-x (PMC5537356; doi:10.1038/s41467-017-00146-x)
Supplement: Supplementary file 2 — Supplementary Software 1 [file 41467_2017_146_MOESM2_ESM.zip › PottsModel/SrcPottsModel/doc/model/Constants.ParameterManager.html]

Constants.ParameterManager


JavaScript is disabled on your browser.


Skip navigation links


- Overview
- Package
- Class
- Use
- Tree
- Deprecated
- Index
- Help

- Prev Class
- Next Class

- Frames
- No Frames

- All Classes

- Summary:
- Nested |
- Field |
- Constr |
- Method

- Detail:
- Field |
- Constr |
- Method


model

## Class Constants.ParameterManager

- java.lang.Object
- - model.Constants.ParameterManager

- Enclosing class:
  :   Constants

  ---

    

  ```
  public class Constants.ParameterManager
  extends java.lang.Object
  ```

  Manages energy information related to this simulation.

- - ### Field Summary

    Fields

    | Modifier and Type | Field and Description |
    | --- | --- |
    | `double` | `areaElasticity` |
    | `double` | `areaStd` |
    | `int` | `decreaseFactorAdhesion` |
    | `int` | `decreaseFactorTemperature` |
    | `int` | `decreaseTime` |
    | `int` | `increaseFactorAdhesion` |
    | `int` | `increaseFactorTemperature` |
    | `int` | `increaseTime` |
    | `int` | `latentTime` |
    | `double` | `maxTemperature` |
    | `double` | `minTemperature` |
    | `Constants.Model` | `model` |
    | `double` | `perimeterElasticity` |
    | `double` | `perimeterStd` |
    | `PixelShape.Type` | `pixelType` |
    | `double` | `targetArea` |
    | `double` | `targetPerimeter` |
    | `double` | `WITHIN_CELL` |
  - ### Constructor Summary

    Constructors

    Constructor and Description

    `ParameterManager(java.util.Properties p)`
  - ### Method Summary

    All Methods Instance Methods Concrete Methods

    | Modifier and Type | Method and Description |
    | --- | --- |
    | `double` | `getCellTypeInteractionEnergy(Cell.CellType t1, Cell.CellType t2)` |
    | `void` | `setCellTypeInteractionEnergies(java.util.HashMap<java.util.EnumSet<Cell.CellType>,java.lang.Double> cellTypeInteractionEnergies)` |
    | `void` | `setInteractionEnergy(Cell.CellType t1, Cell.CellType t2, double n)` |

    - ### Methods inherited from class java.lang.Object

      `equals, getClass, hashCode, notify, notifyAll, toString, wait, wait, wait`

- - ### Field Detail


    - #### pixelType

      ```
      public PixelShape.Type pixelType
      ```


    - #### model

      ```
      public Constants.Model model
      ```


    - #### WITHIN\_CELL

      ```
      public double WITHIN_CELL
      ```


    - #### areaElasticity

      ```
      public double areaElasticity
      ```


    - #### perimeterElasticity

      ```
      public double perimeterElasticity
      ```


    - #### targetArea

      ```
      public double targetArea
      ```


    - #### targetPerimeter

      ```
      public double targetPerimeter
      ```


    - #### areaStd

      ```
      public double areaStd
      ```


    - #### perimeterStd

      ```
      public double perimeterStd
      ```


    - #### latentTime

      ```
      public int latentTime
      ```


    - #### increaseTime

      ```
      public int increaseTime
      ```


    - #### decreaseTime

      ```
      public int decreaseTime
      ```


    - #### minTemperature

      ```
      public double minTemperature
      ```


    - #### maxTemperature

      ```
      public double maxTemperature
      ```


    - #### decreaseFactorTemperature

      ```
      public int decreaseFactorTemperature
      ```


    - #### increaseFactorTemperature

      ```
      public int increaseFactorTemperature
      ```


    - #### decreaseFactorAdhesion

      ```
      public int decreaseFactorAdhesion
      ```


    - #### increaseFactorAdhesion

      ```
      public int increaseFactorAdhesion
      ```
  - ### Constructor Detail


    - #### ParameterManager

      ```
      public ParameterManager(java.util.Properties p)
      ```
  - ### Method Detail


    - #### setCellTypeInteractionEnergies

      ```
      public void setCellTypeInteractionEnergies(java.util.HashMap<java.util.EnumSet<Cell.CellType>,java.lang.Double> cellTypeInteractionEnergies)
      ```


    - #### getCellTypeInteractionEnergy

      ```
      public double getCellTypeInteractionEnergy(Cell.CellType t1,
                                                 Cell.CellType t2)
      ```


    - #### setInteractionEnergy

      ```
      public void setInteractionEnergy(Cell.CellType t1,
                                       Cell.CellType t2,
                                       double n)
      ```


Skip navigation links


- Overview
- Package
- Class
- Use
- Tree
- Deprecated
- Index
- Help

- Prev Class
- Next Class

- Frames
- No Frames

- All Classes

- Summary:
- Nested |
- Field |
- Constr |
- Method

- Detail:
- Field |
- Constr |
- Method
